# Seeing‐good‐gene‐based mate choice: From genes to behavioural preferences

**DOI:** 10.1111/1365-2656.13071

**Published:** 2019-08-15

**Authors:** Li Sun, Tong Zhou, Graham N. Stone, Qiu‐Hong Wan, Sheng‐Guo Fang

**Affiliations:** ^1^ MOE Key Laboratory of Biosystems Homeostasis & Protection, State Conservation Centre for Gene Resources of Endangered Wildlife, College of Life Sciences Zhejiang University Hangzhou China; ^2^ Institute of Evolutionary Biology Edinburgh UK

**Keywords:** conservation, major histocompatibility complex, mate choice, reproductive outputs, visual cues

## Abstract

1. Although vertebrates have been reported to gain higher reproductive outputs by choosing mates, few studies have been conducted on threatened species. However, species recovery should benefit if natural mate choice could improve reproductive output (i.e. pair performance related to offspring number, such as increased clutch size, numbers of fertilized egg and fledglings). We assessed the evidence for major histocompatibility complex (MHC)‐based mate preference in the endangered crested ibis (*Nipponia nippon*) and quantified the impacts of such choice on reproductive output.
2. We tested the hypothesis that crested ibis advertise “good genes” through external traits, by testing whether nuptial plumage characteristics and body morphology mediate mate choice for underlying genetic MHC variation.
3. We found differences between males and females in preferred MHC genotypes, external traits used in mate choice and contributions to reproductive outputs. Females preferred MHC‐heterozygous males, which had darker [i.e. lower total reflectance and ultraviolet (UV) reflectance] nuptial plumage. Males preferred females lacking the DAB*d allele at the MHC class II DAB locus, which had higher average body mass. DAB*d‐free females yielded heavier eggs and more fledglings, while MHC‐heterozygous males contributed to more fertilized eggs and fledglings. Fledging rate was highest when both parents had the preferred MHC genotypes (i.e. MHC‐heterozygous father and DAB*d‐free mother). Comparisons showed that free‐mating wild and semi‐natural pairs yielded more fertilized eggs and more fledglings, with a higher fledging rate, than captive pairs matched artificially based on pedigree.
4. Conservation programmes seldom apply modern research results to population management, which could hinder recovery of threatened species. Our results show that mate choice can play an important role in improving reproductive output, with an example in which an endangered bird selects mates using UV visual capability. Despite the undoubted importance of pedigree‐based matching of mates in conservation programmes, we show that free mating can be a better alternative strategy.

## Graphical abstract

The authors showed a visual cue‐based mate choice in the endangered crested ibis, found higher reproductive outputs in genetically preferred pairs than in genetically nonpreferred pairs and compared reproductive outputs between free‐mating and artificial‐matching pairs. They suggest free mating is worth investigating in conservation programmes.

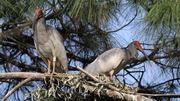

## 1 INTRODUCTION

More than 12.1% of described vertebrates are listed as threatened in the International Union for Conservation of Nature (IUCN) Red List of Threatened Species (IUCN, 2018). Human activity is adding more species to the list at an alarming rate (Butchart et al., 2010), causing ecosystem disruption (Hooper et al., 2012) and negative impacts on human society by dismantling of ecosystem services (Cardinale et al., 2012). Meanwhile, some vertebrates show better reproductive performance by mating with preferred mates (Ihle, Kempenaers, & Forstmeier, 2015; Raveh et al., 2014). It is possible therefore that allowing free mating in conservation programmes, where the primary goal is improved reproductive output, could benefit the recovery of threatened vertebrates.

Most ex situ conservation breeding programmes use artificial pair matching, which excludes natural mate choice. The crested ibis (*Nipponia nippon*) (Pelecaniformes; Threskiornithidae), classified as endangered by the IUCN (IUCN, 2018), has been the focus of dozens of ex situ conservation programmes in recent decades, most of which use artificial matching. However, these populations show breeding problems such as low fertility and fledging rates. The key question we address here is whether free mating, involving natural mate choice, would benefit the recovery of this species by reducing these problems.

We examined whether free‐mating crested ibis show MHC‐based mate choice. We focused on MHC genes as molecular markers for genetic diversity for two reasons. First, they play important roles in the vertebrate immune system, which could strongly influence individual fitness. Second, we have completely analysed the genomic structure of crested ibis MHC genes (Jäger et al., 2007). MHC class I contains five genes, of which only one locus (called UAA hereafter) is functionally polymorphic (i.e. with nonsynonymous mutations between alleles). MHC class II contains four α gene copies (DAA, DBA1, DBA2 and DBA3) and four β gene copies (DAB, DBB1, DBB2 and DBB3), of which only the DAB locus is functionally polymorphic. Class II *α* and *β* genes are arranged alternatively in tandem (i.e. DAA/DAB‐DBA1/DBB1‐DBA2/DBB2‐DBA3/DBB3). Each pair of α and β genes (e.g. DAA/DAB) encodes one kind of MHC protein molecule, called a dyad (Chen et al., 2015). Seven MHC haplotypes [combinations of different alleles at the two functionally polymorphic loci (UAA and DAB)] have been previously identified (Lan, 2014).

Previous research on genetic‐based mate choice in birds has combined quantification of individual genetic diversity and behavioural observation. For example, female scarlet rose finches (*Carpodacus erythrinus*) seek extra‐pair matings less frequently when paired with highly heterozygous males (Promerová et al., 2015). Blue tits also prefer high genetic heterozygosity when they choose mates (GarcãA‐Navas, Ortego, & Sanz, 2009). We explored correlations between mate choice and genetic diversity at MHC genes in crested ibis. Since both males and females are involved in breeding activities (e.g. nest building, hatching and brooding), we examined possible mechanisms in both sexes.

We used genetic information for each MHC class (UAA, DAB) and for overall MHC haplotype to examine three possible mate choice patterns: choice for MHC dissimilarity, choice for MHC heterozygosity and choice for specific MHC alleles (or haplotypes). If free‐mating birds favour MHC‐dissimilar mates, then observed breeding pairs should show higher MHC dissimilarity than expected for random mating. If they prefer mates with high MHC heterozygosity, then observed breeding birds should show higher MHC heterozygosity than randomly selected individuals. Alternatively, if free‐mating birds favour mates with specific MHC alleles, then such alleles should be exhibited at a higher frequency in breeding birds than in randomly selected individuals. To test whether mate choice may be based on genome‐wide (rather than MHC‐specific) genetic diversity, we also examined relationships between reproductive success and genome‐wide genetic diversity estimated with a panel of microsatellites.

Multiple external traits can indicate underlying genotypes and genetic quality in vertebrates. In yellowthroats (*Geothlypis trichas*), MHC diversity is correlated with facial mask size or bib brightness (Dunn, Bollmer, Freeman‐Gallant, & Whittingham, 2013; Whittingham, Freeman‐Gallant, Taff, & Dunn, 2015). Mice prefer mates with dissimilar MHC genotypes, which they detect through the impacts of these genes on body odour (Penn & Potts, 1998). In three‐spined sticklebacks (*Gasterosteus aculeatus*), male breeding coloration is most intense in individuals with maximal MHC class I diversity (Jäger et al., 2007). We therefore hypothesize that vertebrates in general, and crested ibis in particular, could use external traits to assess fitness‐related genetic information and so select a preferred mate for higher reproductive output.

We analysed the relationships between crested ibis MHC variation, external traits and mate choice to explore whether the birds use external traits in mate selection. Adult crested ibises display unique nuptial plumage coloured by a black colorant substance secreted from a patch of black‐pigmented neck skin. Adult birds start to secrete this colorant just before the beginning of each breeding season and transfer it to their feathers by rubbing their necks against their upper backs while bathing in water, resulting in an ash‐black coloration on their neck and back (Wingfield et al., 2000). We asked whether external traits (i.e. nuptial plumage characteristics, body size and body mass) could be used as cues by male and/or female crested ibis for selection of mates with specific MHC genotypes, leading to enhanced reproductive outputs. If assortative mating results in selection of good genes, then P‐P pairs, in which both parents carry MHC genotypes preferred by the opposite sex, should show higher reproductive outputs (expressed as clutch size, egg weight, numbers of fertilized eggs and fledged young) than NP‐NP pairs, in which both parents carry MHC genotypes not preferred by the opposite sex. Further, free‐mating pairs should show higher reproductive outputs than artificially matched pairs, in which natural mate choice was excluded. We tested these hypotheses by comparing numbers of fertilized eggs and fledglings, and fledging rate, between free‐mating wild and semi‐natural pairs and captive artificially matched pairs.

## 2 MATERIALS AND METHODS

### 2.1 Study populations

We studied a wild crested ibis population at Xiazhu Lake (120°2′E, 30°31′N), Zhejiang Province, China, over four years between 2014 and 2017. Wild birds were identifiable by a numbered ring on the tarsus and were sexed based on the sexually dimorphic CHD1 gene (He, Qing, Han, & Ding, 2013) during annual population monitoring. We also collected reproductive data for a captive population and a semi‐natural population at the Deqing Crested Ibis Breeding Centre, also in Zhejiang Province. The semi‐natural population comprised a group in a large enclosure being trained for introduction to the wild. Wild and semi‐natural crested ibises were free to choose mates, while captive crested ibises were artificially matched with a single mate based on pedigree.

### 2.2 Sampling and data collection

We conducted field observations of the wild population in all four years from early February before the birds started to build nests. We captured all observed breeding and nonbreeding wild birds using mist nets and collected blood samples for extraction of genomic DNA (gDNA). We also collected samples of the black colorant (black solid particles adhering to the pigmented skin and surrounding the down feathers) and measured tarsus length, bill length and body mass from 60 males and 39 females. Colourant samples were scraped from the pigmented skin using sterilized wooden toothpicks and quantified using an AvaSpec‐2048 spectrometer (Ocean Optics, Dunedin, FL) equipped with an AvaLight‐DH‐S‐BAL light source. We calculated two reflectance parameters (Montgomerie, 2006): total reflectance (average reflectance from 300 nm to 700 nm) and UV reflectance (average reflectance from 300 nm to 400 nm). These two measures incorporate the visible spectrum of birds and include UV wavelengths known to influence mate choice in other bird species (Bennett, Cuthill, Partridge, & Maier, 1996).

Daily field surveys were conducted when wild couples started to build nests. We recorded the ring numbers of nest‐defending parents and visited wild nests daily to record initial weight of eggs, clutch size and numbers of fertilized eggs and fledglings. Eggs were determined as fertilized or unfertilized through egg candling three days after incubation. A total of 68 wild pairs were observed over four years. To allow comparison between free‐mating and artificially matched pairs, our study incorporated additional data on numbers of fertilized eggs and fledglings for 24 semi‐natural free‐mating pairs and 60 captive artificially matched pairs recorded by the Deqing Crested Ibis Breeding Centre.

### 2.3 Genotyping

We used the phenol–chloroform method (Sambrook, Fritsh, & Maniatis, 1989) to extract gDNA. As described above, crested ibis have two functional polymorphic MHC loci: MHC class I locus UAA (two alleles: UAA*01 and UAA*02) and MHC class II locus DAB (four alleles: DAB*f, DAB*c, DAB*d and DAB*e). We genotyped these two loci using specific primers (Table S1) and determined haplotypes for wild crested ibises as previously described (Lan, Zhou, Wan, & Fang, 2019).

We also genotyped 21 polymorphic microsatellite loci (Table S2) (He, Wan, Fang, & Xi, 2006; Sun, 2018) to capture genome‐wide genetic diversity of the wild, semi‐natural and captive birds. Genetic properties of the microsatellites and MHC haplotypes of wild birds are shown in Table S3. We also calculated the *g*_2_ value (Patrice, Benoit, Frédérique, Vincent, & Jérome, 2007) of the wild population using the R package inbreedR (Stoffel et al., 2016) and found the wild birds to be significantly inbreeding (*p* = .01, Table S4), which implies that our microsatellites reflect individual inbreeding level well. Pairwise Fst values were calculated using FSTAT 2.9.3 (Goudet, 2001) to test for possible genetic differentiation between captive, semi‐natural and wild populations.

### 2.4 Data analyses

#### 2.4.1 MHC parameters

MHC dissimilarity was measured by the number of shared alleles (*S*_xy_), antigen‐binding site (ABS) amino acid evolutionary distance (*D*_xy_), ABS amino acid functional distance (*F*_xy_) and number of different ABS amino acids (*N*_xy_) between spouses (for more details, see Appendix S1). Single locus (UAA and DAB) and haplotype heterozygosities were scored as 1 (heterozygous) or 0 (homozygous). MHC multi‐locus heterozygosity was calculated as the proportion of the two loci that were heterozygous. Specific allele and haplotype frequencies were calculated as proportions in breeding or randomly selected (breeding and nonbreeding) individuals.

#### 2.4.2 Microsatellite parameters

Two genome‐wide heterozygosity metrics—standardized heterozygosity (Coltman, Pilkington, Smith, & Pemberton, 1999) and *d* ^2^ (Coltman & Slate, 2003)—were calculated for 21 polymorphic microsatellite loci in Excel.

#### 2.4.3 Randomization tests

We used the Excel add‐in program RSXL v4.0 to run randomizations in wild free‐mating birds. To assess whether free‐mating birds pair non‐randomly with respect to genetic diversity, we compared observed mean values of MHC and microsatellite indices across pairs with a frequency distribution of mean values generated for 10,000 random pairings drawn from the same set of male and female individuals. Specific steps were as follows: (a) we randomly selected n males/females from all breeding and nonbreeding males/females at the breeding site each year, where n is the number of breeding males/females in that year. (b) We averaged the genetic values of these randomly selected individuals across the four years. (c) We repeated (a) and (b) 10,000 times, resulting in 10,000 random average values. (d) We compared the observed average genetic values of breeding males/females with the 95% confidence intervals (CIs) of the 10,000 random values. The two‐tailed *p*‐value was calculated as twice the proportion of random values that were larger (or smaller) than the observed value. We considered the difference to be significant if the *p*‐value was smaller than .05. When testing for DAB allele frequencies, as we tested four alleles at the same time, we used the Bonferroni correction to adjust our threshold significance level for at least one significant result from .05 to .0125.

### 2.5 Statistical analysis

We used SPSS version 20.0 for Windows (SPSS Inc., Chicago, IL, USA) to perform all analyses. All tests were two‐tailed, and *p* <.05 was considered as significant. A binary logistic regression analysis was performed to investigate the effect of male MHC and microsatellite heterozygosity (as a covariate) on male breeding status. We collapsed bill length and tarsus length to a single measure of body size using principal component analysis (PCA), incorporating the value for the first PCA axis in our analysis. Continuous variables (trait variables and mean egg initial weight) were tested for normality by Kolmogorov–Smirnov tests before parametric tests. The data for all *t* tests in our study satisfied Levene's test of homogeneity of variance. Independent samples *t* tests were employed to compare continuous trait variables (body size, body mass, total reflectance and ultraviolet reflectance) between MHC‐heterozygous and MHC‐homozygous males, and between DAB*d‐carrying and DAB*d‐free females. We used Spearman rank correlation (*r*) to analyse the relationship between MHC multi‐locus heterozygosity and the same set of trait variables.

We used mediation analysis to test whether external visual cues mediate the relationship between MHC variation and breeding status (i.e. the mate choice of the opposite sex), using a bootstrapping method (Preacher & Hayes, 2008). Mediation analysis is widely used by behavioural scientists to identify the means (M) by which an independent variable (X) exerts its influence on the dependent variable (Y). In the present study, M, X and Y represent external traits, MHC variation and breeding status, respectively (i.e. the mate choice of the opposite sex).

Generalized linear modelling (GLM) was employed to examine the impacts of parental MHC types on reproductive outputs. We used MHC multi‐locus heterozygosity to represent paternal MHC heterozygosity to avoid collinearity since the other three measures (DAB, UAA and haplotype heterozygosity) were highly correlated with MHC multi‐locus heterozygosity (Spearman rank correlation coefficient values ranged from .519 between UAA and DAB heterozygosity to .916 between UAA and multi‐locus heterozygosity, all *p*‐values < .01). In addition, since adult age has been reported as relevant to reproductive outputs (Yu, 2007), we added female age and male age as model predictors. Parent birds were divided into three age groups: group 1 included relatively young parent birds between 2 and 3 years old, group 2 included experienced parent birds between 4 and 7 years old, and group 3 included birds than were older than 7 years. We performed Poisson GLMs on number of fertilized eggs and number of fledglings, and a linear GLM on egg initial weight. Because the variance‐to‐mean ratio (VMR) for clutch size is underdispersed (VMR = 0.34), we used a binomial GLM with the maximum clutch size of 5 as the number of trials and the observed clutch size as the number of successes. We used chi‐square tests to compare fledging rate among pairs of different MHC combinations and among pairs of different populations.

## 3 RESULTS

### 3.1 MHC‐based mate preferences in wild crested ibises

**Figure 1:**
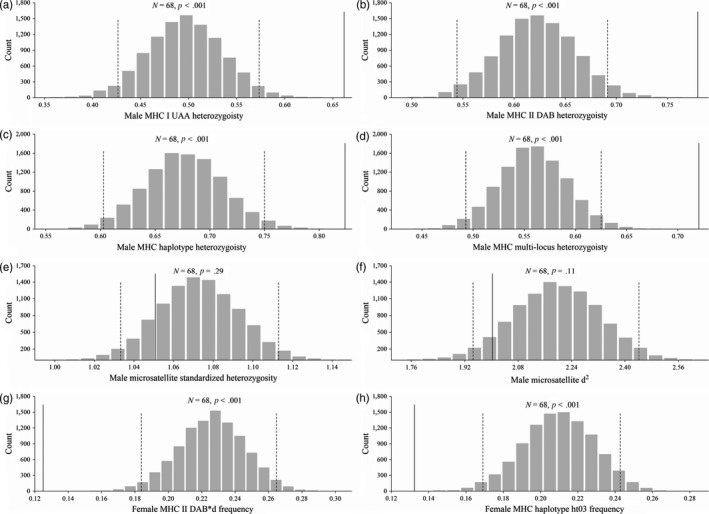
Randomizations of male and female major histocompatibility complex (MHC) types. (a) UAA heterozygosity, (b) DAB heterozygosity, (c) haplotype heterozygosity, (d) MHC multi‐locus heterozygosity, (e) microsatellite standardized heterozygosity and (f) microsatellite d^2^ of breeding males (solid line) compared with the frequency distributions of mean values of random males generated from 10,000 simulations. In addition, (g) DAB*d frequency and (h) ht03 frequency are compared between breeding females (solid line) and the frequency distributions of mean values of random females generated from 10,000 simulations. Two‐tailed 95% confidence intervals are indicated by dashed lines. The numbers of chosen males and females are shown as *N*. *p*‐values are given for each subfigure

There was no evidence for mate selection based on MHC dissimilarity alone in crested ibis: MHC dissimilarity showed no significant difference between observed wild pairs and randomly matched pairs for the same population (Figures S1 and S2). Breeding males had significantly higher MHC heterozygosity at single‐locus [UAA (*p* < .001, Figure 1a), DAB (*p* < .001, Figure 1b)], haplotype (*p* < .001, Figure 1c) and multi‐locus (*p* < .001, Figure 1d) levels than randomly selected males. In contrast, we found no significant difference in genome‐wide microsatellite heterozygosity [standardized heterozygosity (*p* = .29, Figure 1e) and *d* ^2^ (*p* = .11, Figure 1f)] between wild breeding males and randomly selected males. Logistic regression analysis also showed that male breeding status was significantly positively correlated with MHC multi‐locus heterozygosity (slope = 2.53, *SE* = 0.54, *p* < .001) but not with microsatellite heterozygosity (Table S5), suggesting that female preference is specifically for MHC heterozygosity rather than for genome‐wide heterozygosity.

In contrast to males, breeding females did not show higher MHC heterozygosity than randomly selected females (Figure S3) but showed significantly lower DAB*d allele frequency (*p* < .001, Figure 1g) and ht03 haplotype frequency (*p* < .001, Figure 1h).

### 3.2 Visual cues in mate choice

**Figure 2:**
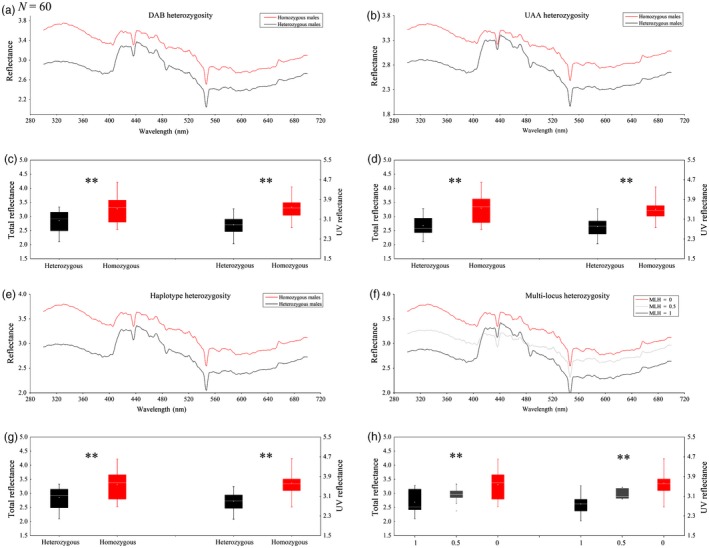
Comparisons of reflectance among male crested ibises carrying different levels of MHC heterozygosity. Each MHC parameter is presented in two figure panels. One shows the average reflectance spectrum for the different genotype groups compared [(a) DAB, (b) UAA, (e) haplotype and (f) MHC multi‐locus heterozygosity], while a second box plot shows the total reflectance and ultraviolet (UV) reflectance for the same groups [(c) DAB, (d) UAA, (g) haplotype and (h) MHC multi‐locus heterozygosity]. *N* indicates the number of samples used for analysis. *p*‐values are given for each box chart (**p < .01)

**Figure 3:**
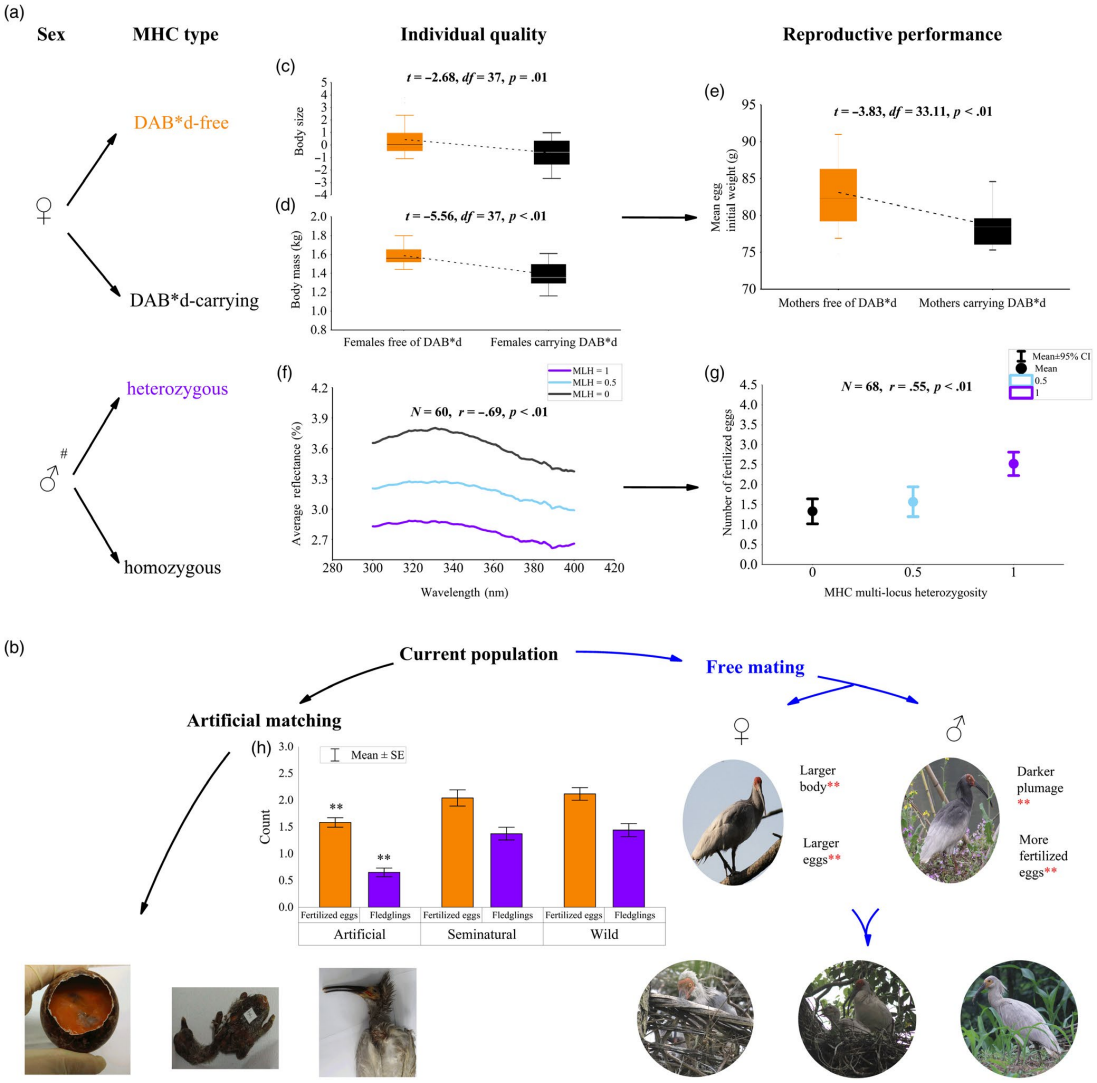
Major histocompatibility complex (MHC)‐based individual performance and comparison between artificial matching and free‐mating crested ibises. (a) Individual quality of (c, d) females and (f) males and reproductive performance of (e) females and (g) males of birds carrying preferred and nonpreferred MHC types. (b) Reproductive outcomes between artificial matching and free mating. The three bottom‐left images show a dead embryo, nestling and fledgling, from left to right. The three bottom‐right images show surviving offspring from parents (the two images above them). The bar graph (h) compares the number of fertilized eggs and fledglings among artificially matched pairs, semi‐natural free‐mating pairs and wild free‐mating pairs. Sample sizes are given as *N*. *p*‐values and corresponding statistics were given for each subfigure (**p < .01). ^#^Comparison of male reflectance spectra and number of fertilized eggs are given at different levels of MLH_MHC_ (MHC multi‐locus heterozygosity)

We further examined whether the external traits of plumage reflectance (total reflectance and UV reflectance) and body morphology (body size and body mass) were correlated with MHC preferences (i.e. male MHC heterozygosity and female DAB*d absence). We found that male MHC heterozygosity was negatively correlated with nuptial plumage colorant reflectance (Figure 2) but not with body morphology (Table S6, Figure S4), while female DAB*d presence was negatively correlated with body morphology (Figure 3c, d) but not with colorant reflectance (Table S7).

We found that DAB heterozygous males secreted significantly darker nuptial plumage colorant, with lower total reflectance (*t* = −4.01, *df* =  58, *p* < .01) and UV reflectance (*t* = −5.71, *df* = 58, *p* < .01) than DAB homozygous males (Figure 2). Similarly, lower reflectance was also found in males heterozygous for UAA (total: *t* = −4.24, *df* = 58, *p* < .01; UV: *t* = −5.79, *df* = 58, *p* < .01), haplotype (total: *t* = −4.30, *df* = 58, *p* < .01; UV: *t* = −5.93, *df* = 58, *p* < .01) and multi‐locus MHC genotype (total: *r* = −.55, *N* = 60, *p* < .01; UV: *r* = −0.69, *N* = 60, *p* < .01). Overall, MHC‐heterozygous males secreted darker (less‐reflecting) plumage colorant than MHC‐homozygous males. Mediation analyses (Table 1) revealed significant effect sizes (i.e. with 95% confidence intervals excluding zero) for the two reflectance parameters combined and for UV reflectance alone, which means that plumage reflectance could mediate the relationship between MHC heterozygosity and breeding status.

**Table 1:** Indirect effects of MHC variation on breeding status through external cues

Independent variables	Mediators	Effect size	Bootstrapping bias‐corrected 95% CI	
			Lower	Higher
Male (*N* = 60)				
DAB heterozygosity	Total	4.57	1.74	12.06
	Total reflectance	−0.20	−2.58	1.96
	UV reflectance	4.77	1.48	11.96
UAA heterozygosity	Total	4.50	1.92	11.69
	Total reflectance	−0.11	−2.60	1.75
	UV reflectance	4.61	1.81	11.02
Haplotype heterozygosity	Total	5.18	2.02	16.37
	Total reflectance	−0.20	−2.85	2.18
	UV reflectance	5.38	1.82	16.07
Multi‐locus heterozygosity	Total	5.97	2.23	20.18
	Total reflectance	−0.19	−3.06	2.42
	UV reflectance	6.16	2.15	18.80
Female (*N* = 39)				
DAB*d presence	Total	−33.05	−67.00	−21.09
	Body size	−2.19	−9.85	0.09
	Body mass	−30.86	−61.93	−18.38

Note

Dependent variable: breeding status; 5,000 bootstrap samples. A separate analysis was conducted for each independent variable.

DAB*d‐free females were larger (*t* = −2.68, *df* =37, *p* = .01, Figure 3c) and heavier (*t* = −5.56, *df* = 37, *p* < .01, Figure 3d) than DAB*d‐carrying females. Mediation analyses (Table 1) revealed significant effect sizes both for two body measurements combined and for body mass alone, indicating that female body morphology could mediate the relationship between DAB*d presence and breeding status.

### 3.3 Offspring viability and parental MHC

Our GLM analyses showed preferred MHC genotypes to be associated with higher measures of reproductive output in both sexes (Table 2). Clutch size and mean initial egg weight were correlated only with maternal factors (Table 2). Mothers with a preferred MHC genotype (i.e. lacking DAB*d) produced eggs with higher mean initial weight (slope = 4.00, *SE* = 1.44, *p* < .01), and more fledglings (slope = 0.82, *SE* = 0.32, *p* < .01). Males with higher MHC heterozygosity sired more fertilized eggs (slope = 0.90, *SE* = 0.40, *p* = .02) and more fledglings (slope = 1.09, *SE* = 0.33, *p* < .01). There was also a significant effect of maternal age: mothers from the middle age group (group 2) produced the largest clutches (slope = 0.69, *SE* = 0.18, *p* < .01) and more fledglings (slope = 1.03, *SE* = 0.39, *p* = .01). We assume the latter relationship to be a by‐product of the relationships between female age and clutch size.

**Table 2:** Correlations between parental age/MHC types and measures of reproductive output

Predictors	Clutch size (*N* = 68)			Number of fertilized eggs^a^ (*N* = 68)			Mean egg initial weight (*N* = 41)			Number of fledglings (*N* = 68)		
	Slope	*SE*	*p*	Slope	*SE*	*p*	Slope	*SE*	*p*	Slope	*SE*	*p*
Maternal DAB*d absence	0.23	0.18	.19	−0.43	0.44	.33	4.00	1.44	**<.01**	0.82	0.32	**.01**
Paternal MHC heterozygosity												
0.5	0.10	0.23	.66	0.27	0.47	.57	−2.51	2.16	.25	0.69	0.41	.09
1	0.23	0.20	.24	0.90	0.40	**.02**	0.31	1.63	.85	1.09	0.33	**<.01**
Maternal age												
Group 2	0.69	0.18	**<.01**	−0.42	0.43	.32	−3.09	1.86	.10	1.03	0.39	**.01**
Group 3	0.08	0.21	.69	−0.06	0.47	.90	−3.93	2.02	.05	0.66	0.42	.12
Paternal age												
Group 2	0.14	0.18	.46	0.08	0.39	.85	2.52	1.71	.14	−0.12	0.39	.75
Group 3	0.25	0.24	.30	−0.24	0.57	.67	2.00	1.95	.30	−0.23	0.53	.67
Year	0.03	0.07	.66	0.04	0.16	.80	−0.04	0.79	.96	0.14	0.14	.33
Constant	−62.10	141.27	.66	−83.73	319.60	.79	163.06	1594.55	.92	−272.71	278.20	.33

Note

A binomial GLM was performed on clutch size. Poisson GLMs were performed on number of fertilized eggs and number of fledglings. A linear GLM was performed on mean initial egg weight. Predictors that are significant are shown in bold.

a Clutch size was used as an offset in the Poisson GLM of the number of fertilized eggs.

**Figure 4:**
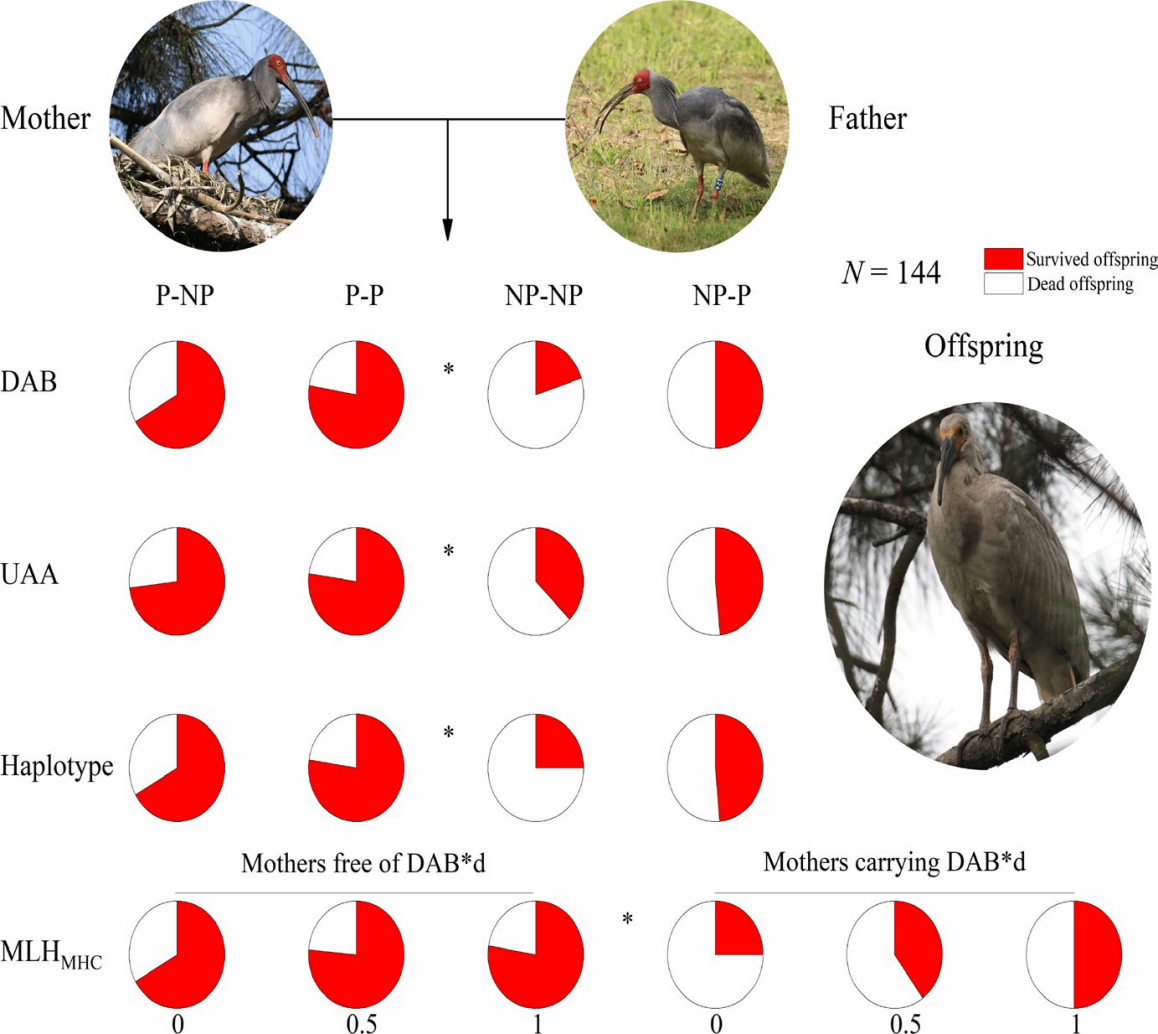
Comparison of proportions of surviving offspring among different pair types in wild crested ibis. The major histocompatibility complex (MHC) loci (DAB and UAA, and haplotype) pie charts are based on the genetic combinations of parents, whether preferred (P) or nonpreferred (NP) with respect to MHC genotype. For mothers, NP and P represent presence and absence, respectively, of the DAB*d (MHC II) allele. For fathers, NP and P represent absence and presence, respectively, of preferred DAB/UAA/haplotype types. The bottom row of MLH_MHC_ (MHC multi‐locus heterozygosity) pie charts is separated based on the genetic combinations of the mother DAB type [with or without DAB*d] and the father MLH_MHC_ values (shown under each pie graph). The total number of offspring used for each analysis is shown as *N*. *p*‐values are calculated using Fisher's exact test between the most preferred (P‐P) and nonpreferred (NP‐NP) parent pairs (*p < 0.05)

Comparison among wild free‐mating pairs with different MHC combinations (Figure 4) showed fledging rate of P‐P pairs (DAB*d‐free mother and MHC‐heterozygous father) to be the highest among all pair types and significantly higher than in NP‐NP pairs (DAB*d‐carrying mother and MHC‐homozygous father) (DAB: *p* < .01; UAA: *p* < .01; haplotype: *p* = .01; multi‐locus heterozygosity: *p* < .01).

### 3.4 Comparison of reproductive outputs between different populations

As shown in Figure 3h, captive artificially matched pairs yielded an average of 1.58 ± 0.70 (mean ± *SE*) fertilized eggs and 0.65 ± 0.63 fledglings, significantly fewer than semi‐natural free‐mating pairs (fertilized eggs: 2.04 ± 0.75, *t* = −2.67, *df* = 82, *p* = .01; fledglings: 1.38 ± 0.58, *t* = −4.86, *df* = 82, *p* < .01) and wild free‐mating pairs (fertilized eggs: 2.12 ± 0.99, *t* = −3.57, *df* = 120.46, *p* < .01; fledglings: 1.44 ± 1.00, *t* = −3.57, *df* = 114.90, *p* < .01). There were no differences between free‐mating pairs in the semi‐natural and wild populations in numbers of fertilized eggs (*t* = −0.34, *df* = 52.79, *p* = .70) or fledglings (*t* = −0.31, *df* = 70.46, *p* = .70). As shown in Figure S6, captive artificially matched pairs yielded a fledging rate of approximately 41.1%, significantly lower than in semi‐natural free‐mating pairs (67.3%, *χ* ^2^ = 8.94, *df* = 1, *p* < .01) and wild free‐mating pairs (68.1%, *χ* ^2^ = 17.06, *df* = 1, *p* < .01). There was no significant difference in fledging rate between free‐mating pairs in semi‐natural and wild populations (*χ* ^2^ = .01, *df* = 1, *p* = .93).

## 4 DISCUSSION

Our results show that crested ibises exhibit mutual MHC‐based mate preferences that may rely on external visual cues, and that correlate positively with enhanced offspring production and viability. Female crested ibises preferred MHC‐heterozygous males, which might be identified by their darker nuptial plumage, and such matings yielded more fertilized eggs and fledglings. Male crested ibises preferred DAB*d‐free females, whose genotypes correlated with larger average body size and mass, and such matings resulted in more and heavier eggs, and more fledglings.

Female preference for male heterozygosity applied specifically to MHC genes and not to genome‐wide microsatellite markers. MHC heterozygosity is expected to confer higher fitness than MHC homozygosity because the former can encode more types of antigen‐presenting MHC molecules. MHC heterozygosity has been linked with higher individual fitness in many studies (Evans & Neff, 2009; Mcclelland, Penn, & Potts, 2003; Penn, Damjanovich, & Potts, 2002; Worley et al., 2010), and heterozygosity is thought to be positively related to male vigour (Mays & Hill, 2004). In crested ibises, males also provided parental care, and female selection of males based on individual fitness should result in mates better able to provide breeding resources (e.g. food, nest material and protection against predators). Our results also showed that male MHC heterozygosity was associated with increases in the number of fertilized eggs and fledglings, indicating that heterozygous males copulated with their mates more frequently and could afford to raise more offspring.

Matches between MHC‐dissimilar parents are expected to generate more heterozygous offspring and thus improve offspring fitness. However, the crested ibises did not show any preference for MHC dissimilarity in our study (Figures S1 and S2). Crested ibis has been reported to have low genetic diversity (Zhang, Fang, & Xi, 2006), and this may mean that selecting mates only on the basis of genetic dissimilarity has limited potential to increase the MHC diversity of offspring.

In contrast to female preference for MHC heterozygosity, male crested ibises preferred females free of a specific MHC class II allele (DAB*d) and haplotype (ht03), indicating that these specific variants might be disadvantageous. Previous work has shown that individuals carrying DAB*d or ht03 only have the two most ancient αβ dyads (i.e. DAA/DAB and DBA3/DBB3) (Chen et al., 2015; Lan, 2014), which could be less well adapted to current environments, while more recent types (i.e. DBA1/DBB1 and DBA2/DBB2) confer superior fitness. In addition, four groups of MHC haplotypes (Figure S5) carry 4, 3, 2 and 1 αβ dyads, respectively, and it is considered advantageous to carry more αβ dyads (i.e. more immune gene loci). However, birds possessing ht03, which carries 2 αβ dyads (i.e. DAA/DAB*d and DBA3/DBB3), rather than ht04 or ht05, which carry only 1 αβ dyad (i.e. DAA/DAB*e), were not preferred in mate choice. We speculate that the MHC protein molecule encoded by DAA/DAB*d might be disadvantageous in terms of antigen resistance capacity compared with DAA/DAB*f, DAA/DAB*c and DAA/DAB*e. Calculation of differences between DAA/DAB sequences and the reference sequence (chicken) showed that functional distance and amino acid difference for DAA/DAB*d were low relative to values for DAA/DAB*f, DAA/DAB*c and DAA/DAB*e (Table S8), probably resulting in lower resistance to pathogens (Dunn et al., 2013; Evans & Neff, 2009; Loiseau et al., 2011; Westerdahl et al., 2005). However, further studies are needed to determine whether DAB*d is associated with susceptibility to certain pathogens.

Vertebrates advertise “good genes” through different kinds of cues (Johansson & Jones, 2010; Ziegler, Kentenich, & Uchanska‐Ziegler, 2005). In mammals and fishes, which have well‐developed olfactory systems, MHC can regulate body odour by encoding volatile acids (Singer, Beauchamp, & Yamazaki, 1997) or changing intestinal flora (Penn & Potts, 1998), making body odour a source of MHC information. Birds do not typically have an acute sense of smell (Jones & Roper, 1997), but they do possess highly developed vision (Eaton, 2005). Relationships between MHC variation, mate choice and individual traits have previously been found in some species (Amundsen & Forsgren, 2001; Sin et al., 2015; von Schantz et al., 1989), and this study shows the mediating role of external traits (i.e. nuptial plumage reflectance and body morphology) in MHC‐based mate choice by the crested ibis. We have connected covariation in MHC variation with crested ibis nuptial plumage reflectance, and deduced the mediating role of this plumage in indicating mate desirability and selection, indicating that avian UV visual capacity is involved in mate choice (Bennett et al., 1996). Although the chemical composition of the nuptial colorant is unknown, its black colour suggests that melanin may be an important component. Lower reflectance from plumage typically indicates a higher proportion of melanin, the amount of which is positively correlated with individual body condition and immunocompetence (Roulin, Dijkstra, Riols, & Ducrest, 2001; Roulin, Jungi, Pfister, & Dijkstra, 2000). Higher production of melanin could be associated with male vigour enhanced by MHC heterozygosity. One possible ecological advantage of the ash‐black plumage is that it could help the birds conceal themselves from predators when staying in the nest or foraging in wetlands. Males with lower‐reflectance plumage are better concealed from predators and thus are more likely to survive to provide breeding resources to females and their progeny.

While we found correlations between MHC variation, external traits and mate choice, we note that such relationships need not be causal. It is possible that such correlations could arise because both MHC and external traits are correlated with individual body condition, which is determined by other factors. Future studies could use manipulative experiments to establish the causal relationships underlying these patterns. For example, manipulation of breeding coloration through dietary supplement or direct experimental intervention, or crossing individuals based on MHC genotypes, could be used in captive populations to illustrate the roles of MHC or breeding coloration in crested ibis breeding more clearly. We also note that increase in offspring number does not guarantee more grandchildren. In the kakapo (*Strigops habroptilus*), for example, well‐fed females produced more offspring, but the heavy sex ratio bias towards sons does not result in extra grandchildren (Sutherland, 2002). Further research involving more than two ibis generations is needed to reveal whether MHC‐based mate preferences lead to more grandchildren. The crested ibis is an excellent model system for such experiments because the genomic organization of its MHC is well‐understood, and we now understand the relationships between important aspects of adult phenotypes, genotypes and reproductive success.

Few studies have documented sexual differences in MHC‐based mate preference, external traits used and their contributions to offspring viability (Viblanc et al., 2016). In crested ibis, although both parents are involved in some breeding activities (e.g. nest building and brooding), other activities differ between the sexes. For example, only females can directly contribute nutrients for embryo development, while males collect most of the nest material (Shi & Cao, 2001). These sex‐specific or sex‐biased reproductive traits are likely to be targeted in mate choice. For example, the DAB*d‐free females preferred by males tend to be larger and heavier, and to lay more and heavier eggs than DAB*d‐carrying females. Similarly, the MHC‐heterozygous males preferred by females are better able to conceal themselves and to find nest‐building resources. In addition, number of fertilized eggs was positively correlated with male MHC heterozygosity, indicating the importance of male vigour in siring more offspring.

We compared reproductive outputs to explore whether pairs from different contexts (i.e. wild, semi‐natural and captive populations) perform differently. Our results showed that wild and semi‐natural free‐mating pairs yielded significantly higher reproductive outputs than captive artificially matched pairs. There may be multiple explanations for poorer performance of captive pairs. First, differences could be due to genetic differences between our three subpopulations. However, *F*_st_ values between all population pairs are <.05, indicating no significant differentiation (Table S9). Further, all three populations were located in Deqing County and experienced very similar environmental conditions (e.g. temperature and photoperiod). Therefore, we consider any impact of subpopulation differentiation unlikely. Second, captive pairs were housed in cages with some exposure to tourists, which could result in stress responses, endocrine disorders and decreased reproductive performance. Third, the captive environment could lack key aspects of the natural environment beneficial to ibis breeding behaviour, resulting in reduced reproductive performance. These are possible subjects for future research. Finally, the underlying mate choice mechanisms we reveal show that the crested ibis, whether in the wild or in enclosed semi‐natural conditions, could experience increased reproductive outputs by freely choosing mates, which is not permitted in captive populations. We suggest that exclusion of free mating could contribute to the poorer reproductive output observed in captive pairs than both semi‐natural and wild pairs.

Conservation breeding programmes are vital tools for the recovery of threatened animals. Although free mating might allow mate choice, for long‐term management of a recovering population, it is still vital that pedigree‐based mating schemes are adopted in captivity to preserve genetic diversity at the expense of marginally slower population growth. Based on our findings, we only suggest that free‐mating breeding management is worth investigating in conservation programmes as it cannot be assumed that artificial matching is the best conservation strategy.

## AUTHORS’ CONTRIBUTIONS

S.‐G.F. conceived and designed this project. L.S. conducted molecular experiments, performed data analysis and drafted the manuscript. T.Z. took the photographs and collected samples. Q.‐H.W. and S.‐G.F. provided the samples and field data. S.‐G.F. provided supervision and he and G.N.S. revised the manuscript. All authors read and approved the final manuscript.

## Data Availability

Data are available from the Dryad Digital Repository: https://doi.org/10.5061/dryad.s75p458 (Sun, Zhou, Stone, Wan, & Fang, 2019).
