# Supplementary material for: Seeing‐good‐gene‐based mate choice: From genes to behavioural preferences
Source: J Anim Ecol. 2019 Aug 15;88(11):1708–19. doi: 10.1111/1365-2656.13071 (PMC6899946; doi:10.1111/1365-2656.13071)
Supplement: Supplementary file 1 [file JANE-88-1708-s001.docx]

**Additional figures**





Fig. S1 Comparison of mean DAB (MHC Ⅱ locus) values between observed pairs and random pairs of crested ibis (*Nipponia nippon*). Allele sharing (S_xy_), amino acid evolutionary distance (D_xy_), amino acid functional distance (F_xy_), and different amino acid number (N_xy_) of observed pairs (solid line) are compared with the frequency distributions of mean values of random pairs generated from 10,000 simulations. Dashed lines indicate two-tailed 95% confidence intervals. The number of observed pairs is shown as *N*. *p*-values are shown for each subfigure.

**

**

Fig. S2 Comparison of mean UAA (MHC Ⅰ locus) values between observed pairs and random pairs of crested ibis (*Nipponia nippon*). Allele sharing (S_xy_), amino acid evolutionary distance (D_xy_), amino acid functional distance (F_xy_) and different amino acid number (N_xy_) of observed pairs (solid lines) were compared with the frequency distributions of mean values of random pairs generated from 10,000 simulations. Dashed lines indicate two-tailed 95% confidence intervals. The number of observed pairs is shown as *N*. *p*-values are shown for each subfigure.


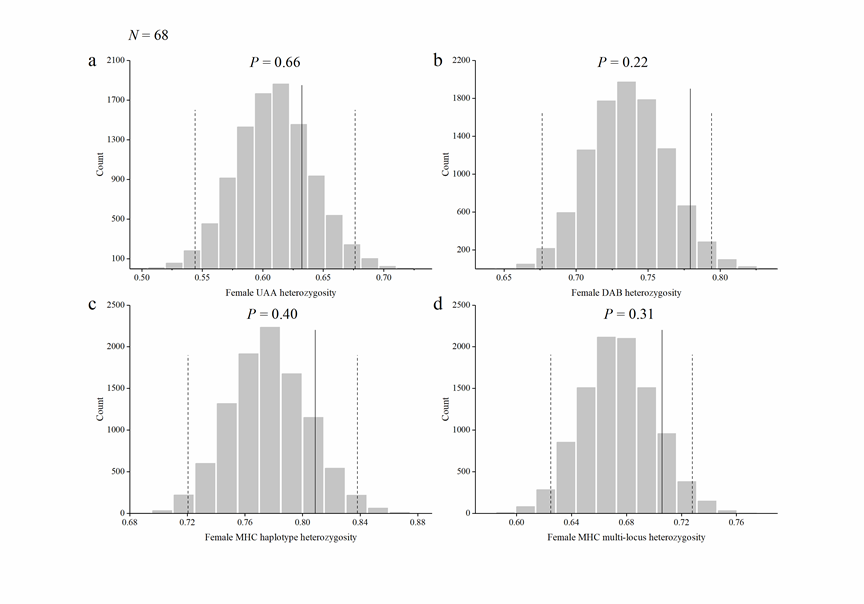


Fig. S3 Comparison of MHC heterozygosity between observed breeding females and randomly selected females. (a) UAA heterozygosity, (b) DAB heterozygosity, (c) haplotype heterozygosity and (d) multi-locus heterozygosity of observed breeding females (solid line) are compared with the frequency distributions of mean values of randomly selected females generated from 10,000 simulations. Dashed lines indicate two-tailed 95% confidence intervals. The number of observed breeding females is shown as *N*. *p*-values are shown for each subfigure.





Fig.S4 Male body measurements in relation to MHC multi-locus heterozygosity. The number of samples is shown as *N*. Pearson correlation *r* and p-values are shown for each subfigure.

Fig. S5 Comparison of the genomic structures among seven MHC haplotypes ([H. Lan, 2014](#_ENREF_3)).





Fig.S6 Comparison of fledging rate among artificially-matched pairs, seminatural free-mating pairs and wild free-mating pairs (**, *p* < 0.01).

**Additional tables.**

Table S1 Primers for two polymorphic MHC loci in crested ibis (*Nipponia* *nippon*)([Hong Lan, Zhou, Wan, & Fang, 2019](#_ENREF_4)). Product size in base pairs (Size) and annealing temperature (*T*_a_) are shown.

| Locus | Location | Primer sequence (5'-3') | Size (bp) | *T*_a_ (℃) |
| --- | --- | --- | --- | --- |
| UAA | Intron1 | ACCTCTGACCTGCCCGGG | 313 | 61 |
|  | Intron2 | ACCCCTGCTCTGCCCCCTGAG |  |  |
|  | Intron2 | CCAGGGGCTCACTGGTATCGC | 345 | 67 |
|  | Intron3 | GGCCGGCACACGCACGATGC |  |  |
| DAB | Intron1  Intron2 | GAGAGCAGCCCGGGGCACA | 359 | 63 |
|  |  | ACGCACTCACGAAATGTGT |  |  |

Table S2 Primers for the 21 polymorphic microsatellite loci from crested ibis (*Nipponia nippon*) ([He, Wan, Fang, & Xi, 2006](#_ENREF_1); [Sun, 2018](#_ENREF_5)). Product size in base pairs (Size) and annealing temperature (*T*_a_) are shown.

| Locus | Repeat motif | Primer sequences (5'-3') | Size (bp) | *T*_a_ (℃) |
| --- | --- | --- | --- | --- |
| NN01 | (CA)_11_ | F: TCAGATAACATTTGTGGGATTG | 194-206 | 58 |
|  |  | R: AGGGCTAGGTTTGGCATT |  |  |
| NN04 | (CA)_2_TA(CA)_11_C(CA)_2_ | F: GTTTCTTCTGTGCCATCC | 211-213 | 57 |
|  |  | R: ATGCCTTGCATTATTGCTT |  |  |
| NN12 | (GT)_19_ | F: TTTCTTCCTCCTGTCAGCTCTTG | 239-243 | 57 |
|  |  | R: GTGCTCTGCACCCTTCACCTTC |  |  |
| NN16 | (GT)_12_ | F: CCAGCCAGTGGGAGTGAAATGC | 237-241 | 58 |
|  |  | R: TGGGATCGTGCTTGGGATCGTG |  |  |
| NN17 | (GT)_11_ | F: CTGGATGTAGGCTTGCTTGGTG | 285-289 | 59 |
|  |  | R: AAGGGGCTGGTTAGTGATAGGG |  |  |
| NN18 | (GT)_20_ | F: TAAAACGAGCCAGACAGTCGCA | 182-196 | 58 |
|  |  | R: GCCCTGAAGCAGTGGTAGGAAG |  |  |
| NN21 | (GA)_12_ | F: CCAGCCTCCTATCCTAATCTAATCG | 175-185 | 57 |
|  |  | R: GAGCCAATCTGTTCCAGTCTCCTT |  |  |
| NN25 | (AAAC)_8_ | F: TCCAGCTACTCACTTCTTTCGG | 166-178 | 57 |
|  |  | R: ATAGATACCCAGGGCATTCAGG |  |  |
| NN26 | (AAAC)_9_ | F: GCACCCAAGACAAAGAGGCAATG | 159-167 | 57 |
|  |  | R: CCTGTTCCAGGGTTTGACCACC |  |  |
| NN5-1 | (ATGAT)_6_ | F, GCATTTGAATTTGTGCAAAATCACCCA | 108-113 | 62 |
|  |  | R, TTCTCCATCCTTGGTGCTGAACAG |  |  |
| NN5-3 | (AAACA)_5_ | F, AGCCTGTGTTTAAAGAGGTAGGCA | 93-98 | 62 |
|  |  | R, AGGTGGAATGAAAAGTAACTGTTTGGA |  |  |
| NN4-3 | (AAAC)_6_ | F, ACCCATTCAGTCACATTACATGGGA | 122-126 | 62 |
|  |  | R, GGAAAAACGTTGCTGTTTCCCCAA |  |  |
| NN4-4 | (CCTT)_5_ | F, TCATACCGCTTCAATGTTCAGCGT | 154-158 | 63 |
|  |  | R, AGAAACAATGGAGGTGCAACAGGT |  |  |
| NN4-5 | (CAAA)_8_ | F, ACTGCCTTAGAGGGACATGTCAGA | 145-149 | 63 |
|  |  | R, AGATTCTCTCTTGAGCTGGTTTTGGG |  |  |
| NN4-8 | (TATC)_5_ | F, ACCAAATGTGTGTCAAAAATACATGGT | 218-222 | 61 |
|  |  | R, AGCCAGAATGTTGAAGCCATAAGG |  |  |
| NN4-12 | (GTTT)_6_ | F, GGCGGGTTTTCAGCTTCTTTGTTT | 147-151 | 63 |
|  |  | R, GCCAATTAACCCAATCTCCAGGCT |  |  |
| NN4-13 | (TTTG)_4_ | F, CAAGCACTGGGGTTTCTTTTTGGT | 151-155 | 63 |
|  |  | R, GTGTGATGTGTGGGTATGCAATGC |  |  |
| NN3-2 | (ATC)_7_ | F, AGCCCAATAAAACTCTTTGCGCAG | 109-112 | 63 |
|  |  | R, TGAGGAACTTGAAAAGAATCCATCCTGT |  |  |
| NN3-3 | (AGG)_4_ | F, CGGCTGAGTAGGAAGAGGAGGATT | 147-150 | 63 |
|  |  | R, CTTGGACCACGTAGAGCTGGAAAG |  |  |
| NN3-5 | (ACA)_6_ | F, CCCTGAAAACTTTTAGGCTGAAAGCT | 120-126 | 62.5 |
|  |  | R, TGTTGGTATCCTTGGTGTGGTGTG |  |  |
| NN3-7 | (ACA)_5_ | F, CTGCTTGCAAAGAACGCTTTTTGC | 141-144 | 63 |
|  |  | R, ATTCCTGGGGTTTATTGCCAAGGC |  |  |

Table S3. Genetic properties of 21 microsatellite loci and MHC haplotypes of the wild crested ibises. Expected heterozygosity is under the assumption of Hardy-Weinberg equilibrium, while Observed heterozygosity is by direct count.

| Locus | Number of alleles | Observed heterozygosity | Expected heterozygosity |
| --- | --- | --- | --- |
| NN01 | 3 | 0.546 | 0.566 |
| NN04 | 2 | 0.311 | 0.366 |
| NN12 | 3 | 0.613 | 0.646 |
| NN16 | 2 | 0.303 | 0.313 |
| NN17 | 2 | 0.370 | 0.395 |
| NN18 | 3 | 0.593 | 0.646 |
| NN21 | 2 | 0.449 | 0.494 |
| NN25 | 2 | 0.445 | 0.482 |
| NN26 | 2 | 0.244 | 0.264 |
| NN5-1 | 2 | 0.271 | 0.294 |
| NN5-3 | 2 | 0.154 | 0.171 |
| NN4-3 | 2 | 0.492 | 0.497 |
| NN4-4 | 2 | 0.483 | 0.502 |
| NN4-5 | 2 | 0.453 | 0.465 |
| NN4-8 | 2 | 0.376 | 0.421 |
| NN4-12 | 2 | 0.437 | 0.486 |
| NN4-13 | 2 | 0.176 | 0.228 |
| NN3-2 | 2 | 0.219 | 0.236 |
| NN3-3 | 2 | 0.500 | 0.502 |
| NN3-5 | 2 | 0.475 | 0.478 |
| NN3-7 | 2 | 0.454 | 0.486 |
| Haplotype | 7 | 0.731 | 0.765 |

Table S4 *g*_2_ value and 95% confidence intervals, standard errors and *P*-value for wild crested ibises genotyped at 21 microsatellites.

|  | *g*_2_ | Lower CI | Upper CI | S.E. | *P*-value |
| --- | --- | --- | --- | --- | --- |
| Microsatellites | 0.022 | 0.001 | 0.041 | 0.010 | **0.013** |

Predictors that are significant are shown in bold.

Table S5 Effects of MHC and microsatellite heterozygosity on male breeding.

|  | Slope | S.E. | df | *P-*value |
| --- | --- | --- | --- | --- |
| MHC multi-locus heterozygosity | 2.47 | 0.54 | 1 | **<0.001** |
| Microsatellite heterozygosity^*^ | -1.04 | 0.99 | 1 | 0.29 |
| Year | 0.08 | 0.21 |  | 0.71 |
| Constant | -157.86 | 417.93 | 1 | 0.71 |

^*^ Microsatellite heterozygosity is represented by microsatellite standardized heterozygosity.

Predictors that are significant are shown in bold.

Table S6 Comparisons of body measurements between MHC heterozygous and homozygous male crested ibis.

|  | Heterozygotes | Homozygotes | *t* | *df* | *P* |
| --- | --- | --- | --- | --- | --- |
| DAB |  |  |  |  |  |
| Bill length(mm) | 173.23±4.21 | 171.96±3.42 | 1.20 | 58 | 0.24 |
| Tarsus length(mm) | 72.34±2.97 | 71.26±3.80 | 1.23 | 58 | 0.22 |
| Body mass (kg) | 1.74±0.09 | 1.71±0.05 | 1.24 | 58 | 0.22 |
| UAA |  |  |  |  |  |
| Bill length(mm) | 173.17±4.52 | 172.32±3.27 | 0.83 | 58 | 0.41 |
| Tarsus length(mm) | 72.32±2.94 | 71.55±3.68 | 0.90 | 58 | 0.37 |
| Body mass (kg) | 1.74±0.09 | 1.72±0.05 | 1.45 | 58 | 0.15 |
| Haplotype |  |  |  |  |  |
| Bill length(mm) | 173.11±4.18 | 172.06±3.45 | 0.98 | 58 | 0.33 |
| Tarsus length(mm) | 72.24±2.94 | 71.37±3.97 | 0.95 | 58 | 0.34 |
| Body mass (kg) | 1.74±0.09 | 1.71±0.05 | 1.14 | 58 | 0.26 |

Table S7 Comparison of nuptial plumage brightness between DAB*d-carrying and DAB*d-free female crested ibises.

|  | DAB*d-carrying females | DAB*d-free females | *t* | *df* | *P* |
| --- | --- | --- | --- | --- | --- |
| Total brightness | 2.91±0.69 | 3.27±0.95 | -1.31 | 37 | 0.20 |
| UV brightness | 3.11±0.89 | 3.53±1.06 | -1.32 | 37 | 0.20 |

Table S8 Functional distances and amino acid differences between crested ibis MHC Ⅱ PBR amino acid sequences and reference sequence.

| Crested ibis αβ PBR | Functional distances | Amino acid differences |
| --- | --- | --- |
| DA αβ |  |  |
| DAA/DAB*f | 1.88 | 73 |
| DAA/DAB*c | 1.81 | 72 |
| DAA/DAB*d | 1.78 | 71 |
| DAA/DAB*e | 1.92 | 74 |
| DB αβ |  |  |
| DBA/DBB1 | 2.21 | 79 |
| DBA/DBB2 | 2.11 | 75 |

Reference sequence: chicken MHC Ⅱ αβ PBR amino acid sequence.

Table S9 Pairwise multi-locus estimates of Fst in crested ibis populations.

|  | Wild | Captive | Seminatural |
| --- | --- | --- | --- |
| Wild |  | 0.0043 | 0.0004 |
| Captive | 0.0043 |  | 0.0015 |
| Seminatural | 0.0004 | 0.0015 |  |

**Nonessential methods**

**MHC similarity parameters calculation.**

To determine if a preference for MHC dissimilarity existed, we compared the number of shared alleles (S_xy_), the number of different amino acids (N_xy_), the amino acid evolutionary distance (D_xy_), and the functional distance (Z_xy_) of antigen binding sites between observed parent pairs with the frequency distribution of those values of randomly selected pairs. S_xy_ indicated the number of alleles shared by parents. D_xy_ was calculated with the following formula:

D_xy_=D_ab_+D_aB_+D_Ab_+D_AB_

where Aa was the genotype of individual x, and Bb was the genotype of individual y. Evolutionary distance between alleles were calculated using MEGA 5.0. For Z_xy_, five z-descriptors, including hydrophobicity (z1), steric bulk (z2), polarity (z3), and electronic effects (z4 and z5), were used to describe the physicochemical property and function of each amino acid ([Huchard, Weill, Cowlishaw, Raymond, & Knapp, 2008](#_ENREF_2)), and a matrix was constructed in Excel to calculate the Euclidean distance between alleles. Z_xy_ between parent pairs was calculated using the same formula as for D_xy_.

References

He, L. P., Wan, Q. H., Fang, S. G., & Xi, Y. M. (2006). Development of novel microsatellite loci and assessment of genetic diversity in the endangered crested ibis, Nipponia nippon. *Conservation Genetics, 7*(1), 157-160.

Huchard, E., Weill, M., Cowlishaw, G., Raymond, M., & Knapp, L. A. (2008). Polymorphism, haplotype composition, and selection in the MHC-DRB of wild baboons. *Immunogenetics, 60*(10), 585-598.

Lan, H. (2014). *Analysis of multilocus haplotype of MHC class Ⅰ and Ⅱ genes and adaptive evolution for crested ibis (in chinese).* (Doctor D. Sc. Thesis), Zhejiang University.

Lan, H., Zhou, T., Wan, Q.-H., & Fang, S.-G. (2019). Genetic diversity and differentiation at structurally varying MHC haplotypes and microsatellites in bottlenecked populations of endangered crested ibis. *Cells, 8*(4), 377.

Sun, L. (2018). *Mate choice pattern of crested ibis (in chinese).* (Doctor D. Sc. Thesis), Zhejiang University.
